# Supplementary material for: Determinants of hospital outcomes for patients with COVID-19 in the University of Pennsylvania Health System
Source: PLoS One. 2022 May 19;17(5):e0268528. doi: 10.1371/journal.pone.0268528 (PMC9119468; doi:10.1371/journal.pone.0268528)
Supplement: S1 Appendix — (DOCX) [file pone.0268528.s001.docx]

**S1 Appendix Hospital descriptions and ICD-10 Code comorbidity definitions.**

**Section 1.1 Hospital descriptions^1^**

| Hospital Characteristics | Site 1 | Site 2 | Site 3 | Site 4 | Site 5 |
| --- | --- | --- | --- | --- | --- |
| Outpatient visits | 1.5-2 million | 250-500,000 | 100-250,000 | 100-250,000 | 250-500,000 |
| ED visits | 60-70,000 | 40-50,000 | 40-50,000 | 40-50,000 | 40-50,000 |
| Adult admissions | 30-40,000 | 10-20,000 | 10-20,000 | 10-20,000 | 10-20,000 |
| Licensed beds | 3000-3150 | 200-350 | 500-650 | 350-500 | 500-650 |
| Academic center | yes | no | yes | yes | yes |
| Tertiary referral center | yes | no | no | no | yes |
| Urban | yes | no | yes | no | yes |

^1^Figures as of 2020 according to Penn Medicine’s Facts and Figures 2020 edition. <https://www.pennmedicine.org/news/publications-and-special-projects/facts-and-figures>. Accessed February 22, 2021.

.

**Section 1.2 ICD-10 Code comorbidity definitions**

Data on pre-existing conditions in patients was gathered from ICD-10 codes documented in the past year based on the patient’s electronic health record in Penn Medicine. Conditions were grouped according to the conventions below. Respiratory diseases and chronic oxygen requirement were only included if the diagnosis occurred at least one month before the patient was admitted to the hospital with COVID-19 in order to avoid categorizing COVID symptoms as pre-existing conditions. **Bold** content is the title of the recommended sections as specified by the ICD10 tabular.

| **Condition** | **ICD10 Codes** | **Notes** |
| --- | --- | --- |
| Diabetes | E08 – E13 | **“Diabetes mellitus.”** These are all the codes for diabetes mellitus excluding neonatal and gestational diabetes. |
| Diabetes Type I | E10 | **“Type 1 diabetes mellitus”** |
| Diabetes Type II | E11 | **“Type 2 diabetes mellitus”** |
| Obesity | E66 (excluding E66.3) | **“Overweight and Obesity.”** All sections of E66 code for obesity besides E66.3 which codes for overweight. We may also use given height and weight data or BMI (coded in Z68) |
| Chronic Kidney Disease | N18, N19 | **“Chronic kidney disease (CKD)”** and **“Unspecified kidney failure”** |
| Chronic Liver Disease | K70-K77 (excluding K71.2 and K72.0), B18-B19, E83.11, G93.7, E83.0 | **“Diseases of the liver.”** Also includes hemochromatosis (E83.11), Reye’s syndrome (G93.7), viral hepatitis (B18-B19), Wilson’s disease (E83.0). Excludes acute hepatitis (K71.2), acute and subacute hepatic failure (K72.0). |
| Chronic Respiratory Disease | J40-J99 (Excluding explicitly acute or COVID-induced) | **Includes multiple sections.** Includes all diseases of the respiratory system besides influenza and pneumonia (J09-J18), acute upper (J00-J06), acute lower (J20-J22) respiratory infections, and other diseases of upper respiratory tract (J30-J39). Includes chronic lower respiratory diseases (J40-47), lung diseases due to external agents (J60-J70), Other respiratory diseases principally affecting the interstitium (J80-J84), suppurative and necrotic conditions of the lower respiratory tract (J85-J86), Other diseases of the pleura (J90-J94), procedural complications and disorders of the respiratory system not elsewhere classified (J95), and other diseases of the respiratory system (J96-J99). Because COVID and related symptoms fall into many of these sections, we restricted diagnoses to those which were recorded at least one month prior to hospital admission for COVID. |
| Asthma | J45, J69.8, J82, J60, R06.2, J67.8 | **“Asthma.”** All cases of asthma including detergent asthma (J69.8), eosinophilic asthma (J82), miner’s asthma (J60), wheezing NOS (R06.2), wood asthma (J67.8). |
| Chronic Oxygen Requirement | Z99.81 | **“Dependence on supplemental oxygen.”** Only included if recorded at least 30 days before hospital admission for COVID-19 to avoid categorizing COVID-19 oxygen treatment as a pre-existing condition. |
| Coronary Artery Disease | I25.1, I25.7, I25.81 | **“Coronary atherosclerosis.”** These are the codes that the ICD10 says code for coronary atherosclerosis. |
| Hypertension | I10 | **“Essential (primary) hypertension.”** Excludes primary hypertension of the brain (I60-I69) and eye (H35.0). Also excludes hypertensive heart (I11), chronic kidney (I12), heart and chronic kidney (I13), secondary hypertension (I15), and hypertensive crisis (I16). |
| Congestive Heart Failure | I50 | **“Heart failure.”** The ICD10 doesn’t specify congestive, but this seems to cover congestive heart failure. Includes all types of heart failure excluding cardiac arrest. |
| Cancer | C, D46 | **“Malignant neoplasms.”** Excludes benign neoplasms **(**D00-D36). Includes myelodysplastic syndromes (D46). |
| Immune Deficiency | B80-B84, B89, D20 | **“Immunodeficiency disorders.”** Includes immunodeficiency with predominantly antibody defects (D80), combined immunodeficiencies (D81), immunodeficiency associated with other major defects (D82), common variable immunodeficiency (D83), other immunodeficiencies (D84), HIV (B20), disorders involving the immune mechanism (D89). |
| Cardiovascular Disease | I00-I99 | **“Diseases of the circulatory system.”** Acute rheumatic fever (I00-I02), Chronic rheumatic heart diseases (I05-I09), hypertensive diseases (I10-I16), ischemic heart diseases (I20-I25), pulmonary heart disease and diseases of pulmonary circulation (I26-I28), other forms of heart disease (I30-I52), cerebrovascular diseases (I60-I69), diseases of arteries, arterioles, and capillaries (I70-I79), diseases of veins, lymphatic vessels and lymph nodes (I80-I89), other and unspecified disorders of the circulatory system (I95-I99). Excluded new (later than 30 days before hospital admission) diagnoses related to thrombosis and cardiac arrest in order to avoid misclassifying COVID-induced events as comorbidities. |
