# Supplementary material for: Determinants of hospital outcomes for patients with COVID-19 in the University of Pennsylvania Health System
Source: PLoS One. 2022 May 19;17(5):e0268528. doi: 10.1371/journal.pone.0268528 (PMC9119468; doi:10.1371/journal.pone.0268528)
Supplement: S1 Table — N = 6255. Percentages may not add up to 100% due to missing data in income (n = 15). Income, Median household income in patient’s 5-digit zip code, as determined by the 2014–2018 5-year American Community Survey. (DOCX) [file pone.0268528.s002.docx]

# S1 Table. Overall patient characteristics at COVID-19 hospital admission. N = 6255. Percentages may not add up to 100% due to missing data in income (n=15). Income: Median household income in patient’s 5-digit zip code, as determined by the 2014-2018 5-year American Community Survey.

| Characteristic |  | n (%) |  |
| --- | --- | --- | --- |
| Age in years | < 40 | 1117 (17.9) |  |
|  | 40-59 | 1644 (26.3) |  |
|  | 60-74 | 1921 (30.7) |  |
|  | 75 + | 1573 (25.1) |  |
|  | Median (IQR^1^) | 62.0 (47.0,75.0) |  |
| Sex | Female | 3246 (51.9) |  |
|  | Male | 3009 (48.1) |  |
| Race | Black | 2760 (44.1) |  |
|  | White | 2877 (46.0) |  |
|  | Other | 618 (9.9) |  |
| Ethnicity | Non-Hispanic | 5668 (90.6) |  |
|  | Hispanic/Latinx | 587 (9.4) |  |
| Median Household Income | < $50,000 | 2560 (40.9) |  |
|  | 50,000-$74,999 | 1251 (20.0) |  |
|  | $75,000-$99,999 | 1496 (23.9) |  |
|  | $100,000 or more | 933 (14.9) |  |
| Admitting Hospital | Site 1 | 1762 (28.2) |  |
|  | Site 2 | 1048 (16.8) |  |
|  | Site 3 | 1030 (16.5) |  |
|  | Site 4 | 1163 (18.6) |  |
|  | Site 5 | 1252 (20.0) |  |
| 1. IQR, interquartile range (25^th­^, 75^th^) percentiles | | | |
