# Supplementary material for: Determinants of hospital outcomes for patients with COVID-19 in the University of Pennsylvania Health System
Source: PLoS One. 2022 May 19;17(5):e0268528. doi: 10.1371/journal.pone.0268528 (PMC9119468; doi:10.1371/journal.pone.0268528)
Supplement: S2 Table — N = 6255. Percentages may not add up to 100% due to missing data in median household income or rounding elsewhere. P-values were for the chi-squared test. Comorbidites included diabetes, cardiovascular disease, respiratory disease, kidney disease, liver disease, immune deficiency, and chronic oxygen requirement. See S1 Appendix for more details. (DOCX) [file pone.0268528.s003.docx]

**S2 Table Comorbidity at COVID-19 hospital admission by race.** N=6255. Percentages may not add up to 100% due to missing data in median household income or rounding elsewhere. P-values from chi-squared test. Any ICD-10 comorbidity included diabetes, cardiovascular disease, respiratory disease, kidney disease, liver disease, immune deficiency, and chronic oxygen requirement. See Supplemental S1 Appendix for more details.

| Condition |  | White (N = 2877) | Black (N = 2760) | Other (N = 618) | P-Value |
| --- | --- | --- | --- | --- | --- |
| Any ICD-10 comorbidity |  | 1627 (56.6) | 1967 (71.3) | 298 (48.2) | < 0.001 |
| Diabetes |  | 386 (13.4) | 789 (28.6) | 65 (10.5) | < 0.001 |
| Kidney disease |  | 223 (7.8) | 515 (18.7) | 41 (6.6) | < 0.001 |
| Liver disease |  | 94 (3.3) | 104 (3.8) | 13 (2.1) | 0.107 |
| Respiratory disease |  | 398 (13.8) | 556 (20.1) | 59 (9.5) | < 0.001 |
| Cardiovascular disease |  | 1353 (47.0) | 1659 (60.1) | 240 (38.8) | < 0.001 |
| Heart failure |  | 306 (10.6) | 443 (16.1) | 65 (10.5) | < 0.001 |
| Cancer |  | 319 (11.1) | 273 (9.9) | 32 (5.2) | < 0.001 |
| ICU at admission |  | 391 (13.6) | 420 (15.2) | 116 (18.8) | 0.003 |
| Age in years | < 40 | 415 (14.4) | 589 (21.3) | 113 (18.3) | < 0.001 |
|  | 40-59 | 663 (23.0) | 811 (29.4) | 170 (27.5) |  |
|  | 60-74 | 893 (31.0) | 830 (30.1) | 198 (22.2) |  |
|  | 75 + | 906 (31.5) | 530 (19.2) | 137 (22.2) |  |
| Income | <$50K | 393 (13.7) | 1997 (72.4) | 170 (27.5) | < 0.001 |
|  | $50K-$75K | 682 (23.7) | 428 (15.5) | 141 (22.8) |  |
|  | $75K+ | 1795 (62.4) | 329 (11.9) | 305 (49.4) |  |
| Body mass index (BMI) | Normal (18.5 ≤ BMI < 25) | 675 (23.5) | 526 (19.1) | 194 (31.4) | < 0.001 |
|  | Obese (BMI ≥ 30) | 1166 (40.5) | 1410 (51.1) | 191 (30.9) | < 0.001 |
|  | Overweight (25 ≤ BMI < 30) | 888 (30.9) | 691 (25.0) | 205 (33.2) |  |
|  | Underweight (BMI< 18.5) | 93 (3.2) | 72 (2.6) | 11 (1.8) |  |
|  | Missing | 55 (1.9) | 61 (2.2) | 17 (2.8) |  |
|  | | | | | |
