# Supplementary material for: Determinants of hospital outcomes for patients with COVID-19 in the University of Pennsylvania Health System
Source: PLoS One. 2022 May 19;17(5):e0268528. doi: 10.1371/journal.pone.0268528 (PMC9119468; doi:10.1371/journal.pone.0268528)
Supplement: S3 Table — N = 6255. Percentages may not add up to 100% due to missing data in median household income or rounding elsewhere. P-values were for the chi-squared test. Any ICD-10 comorbidity included diabetes, cardiovascular disease, respiratory disease, kidney disease, liver disease, immune deficiency, and chronic oxygen requirement. See S1 Appendix for more details. (DOCX) [file pone.0268528.s004.docx]

**S3 Table Comorbidity at COVID-19 hospital admission by Hispanic/Latinx ethnicity.** N=6255. Percentages may not add up to 100% due to missing data in median household income or rounding elsewhere. P-values were for the chi-squared test. Any ICD-10 comorbidity included diabetes, cardiovascular disease, respiratory disease, kidney disease, liver disease, immune deficiency, and chronic oxygen requirement. See Supplemental S1 Appendix for more details.

| Condition |  | Hispanic/Latinx (N = 587) | Other (N = 5668) | p-Value^2^ |
| --- | --- | --- | --- | --- |
| Any ICD-10 comorbidity |  | 251 (42.8) | 3641 (64.2) | < 0.001 |
| Diabetes |  | 95 (16.2) | 1145 (20.2) | 0.023 |
| Kidney disease |  | 23 (3.9) | 756 (13.3) | < 0.001 |
| Liver disease |  | 21 (3.6) | 190 (3.4) | 0.867 |
| Respiratory disease |  | 46 (7.8) | 967 (17.1) | < 0.001 |
| Cardiovascular disease |  | 184 (31.3) | 3068 (54.1) | < 0.001 |
| Heart failure |  | 24 (4.1) | 790 (13.9) | < 0.001 |
| Cancer |  | 17 (2.9) | 607 (10.7) | < 0.001 |
| ICU at admission |  | 80 (13.6) | 847 (14.9) | 0.428 |
| Age | < 40 | 225 (38.3) | 892 (15.7) | < 0.001 |
|  | 40-59 | 191 (32.5) | 1453 (25.6) |  |
|  | 60-74 | 115 (9.5) | 1806 (26.8) |  |
|  | 75 + | 56 (9.5) | 1517 (26.8) |  |
| Median household income | <$50K | 166 (28.3) | 2394 (42.2) | < 0.001 |
|  | $50K-$75K | 138 (23.5) | 1113 (19.6) |  |
|  | $75K+ | 282 (48.0) | 2147 (37.9) |  |
| Body mass index (BMI) | Normal (18.5 ≤ BMI < 25) | 83 (14.1) | 1312 (23.1) | < 0.001 |
|  | Obese (BMI ≥ 30) | 274 (46.7) | 2493 (44.0) | < 0.001 |
|  | Overweight (25 ≤ BMI < 30) | 202 (34.4) | 1582 (27.9) |  |
|  | Underweight (BMI< 18.5) | 8 (1.4) | 168 (3.0) |  |
|  | Missing | 20 (3.4) | 113 (2.0) |  |
