# Supplementary figures and images for: Determinants of hospital outcomes for patients with COVID-19 in the University of Pennsylvania Health System
Source: PLoS One. 2022 May 19;17(5):e0268528. doi: 10.1371/journal.pone.0268528 (PMC9119468; doi:10.1371/journal.pone.0268528)

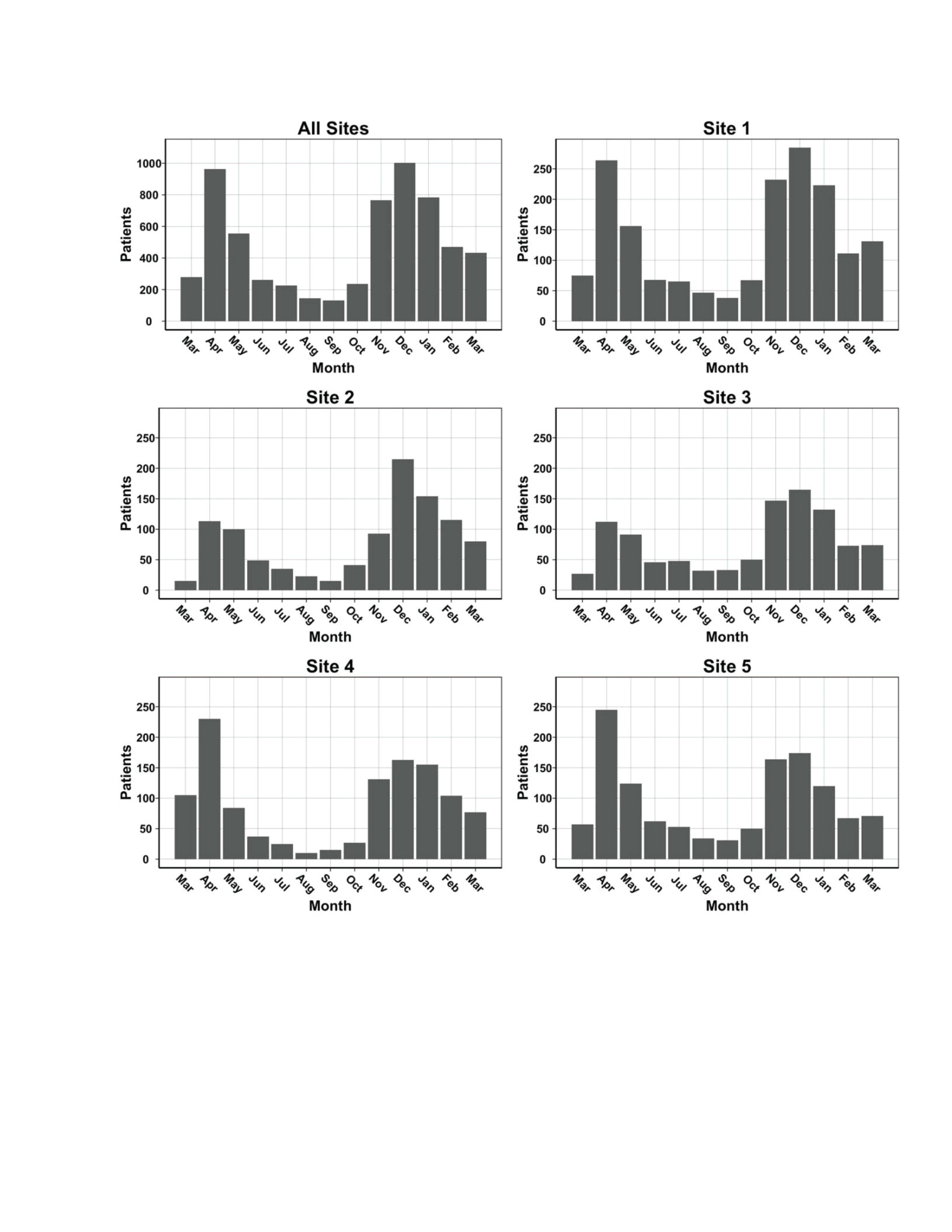

Supplement: S1 Fig — Site 1 (n = 1762), Site 2 (n = 1048), Site 3 (n = 1030), Site 4 (n = 1163), Site 5 (n = 1252) are unique hospitals in the University of Pennsylvania Health System. (TIF) [file pone.0268528.s005.tif]

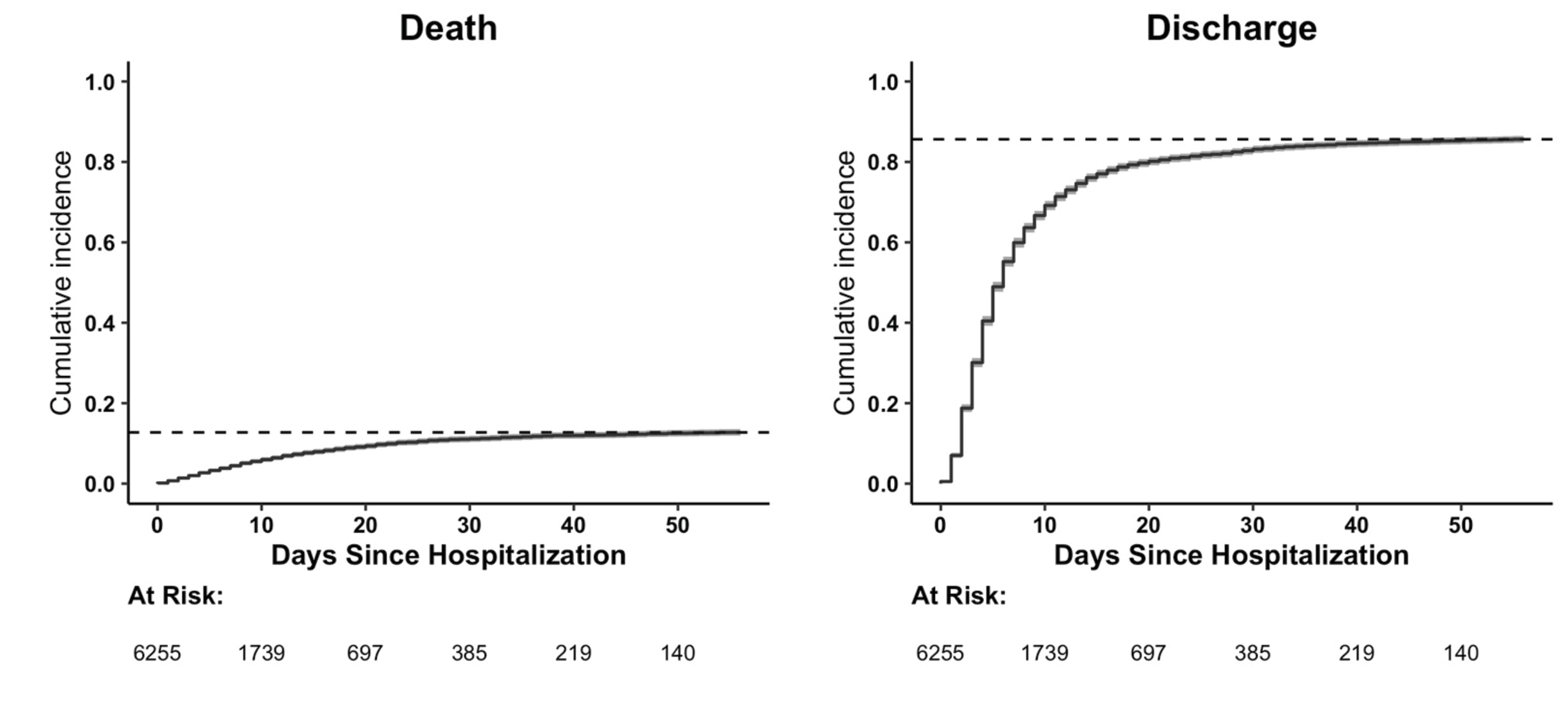

Supplement: S2 Fig — N = 6255. (TIF) [file pone.0268528.s006.tif]

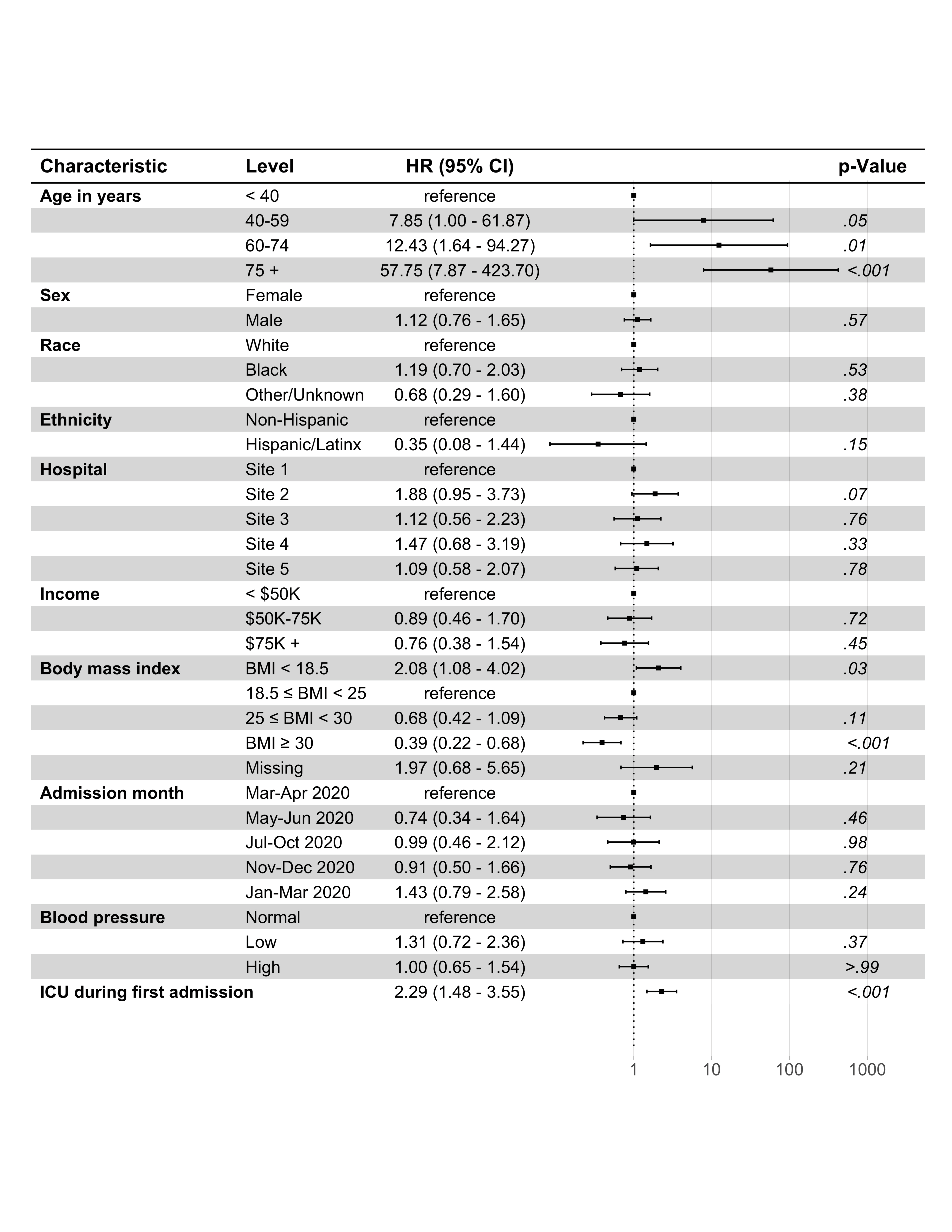

Supplement: S3 Fig — N = 6233. 22 observations deleted due to missingness in zip code (15) and blood pressure (7). Income: Median Household Income in patient’s 5-digit zip code, as determined by the 2014–2018 5-year American Community Survey; CI: Confidence interval; Site 1 (n = 1762), Site 2 (n = 1048), Site 3 (n = 1030), Site 4 (n = 1163), Site 5 (n = 1252) are unique hospitals in the University of Pennsylvania Health System. (TIF) [file pone.0268528.s007.tif]

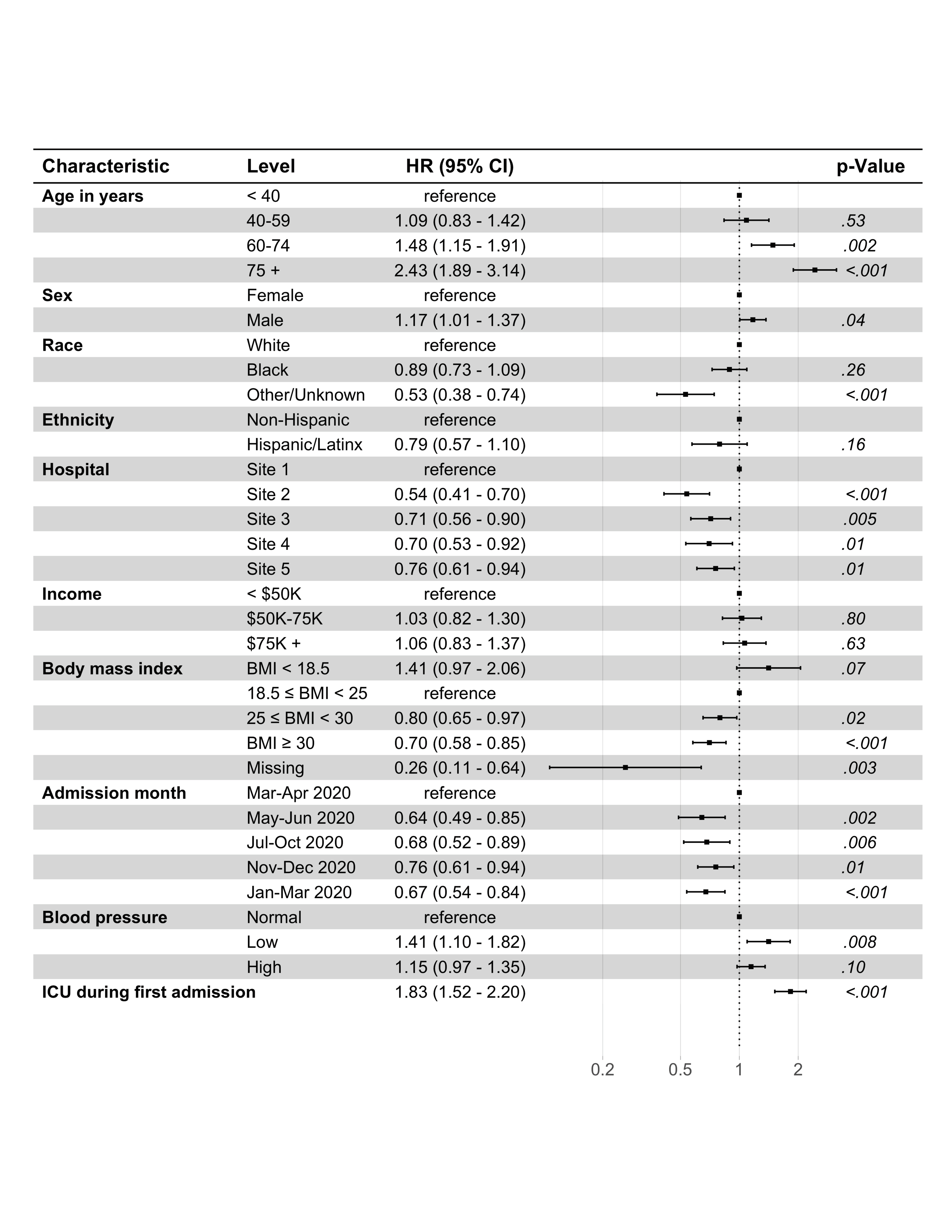

Supplement: S4 Fig — N = 6233. 22 observations deleted due to missingness in zip code (15) and blood pressure (7). Income: Median Household Income in patient’s 5-digit zip code, as determined by the 2014–2018 5-year American Community Survey; CI: Confidence interval; Site 1 (n = 1762), Site 2 (n = 1048), Site 3 (n = 1030), Site 4 (n = 1163), Site 5 (n = 1252) are unique hospitals in the University of Pennsylvania Health System. (TIF) [file pone.0268528.s008.tif]

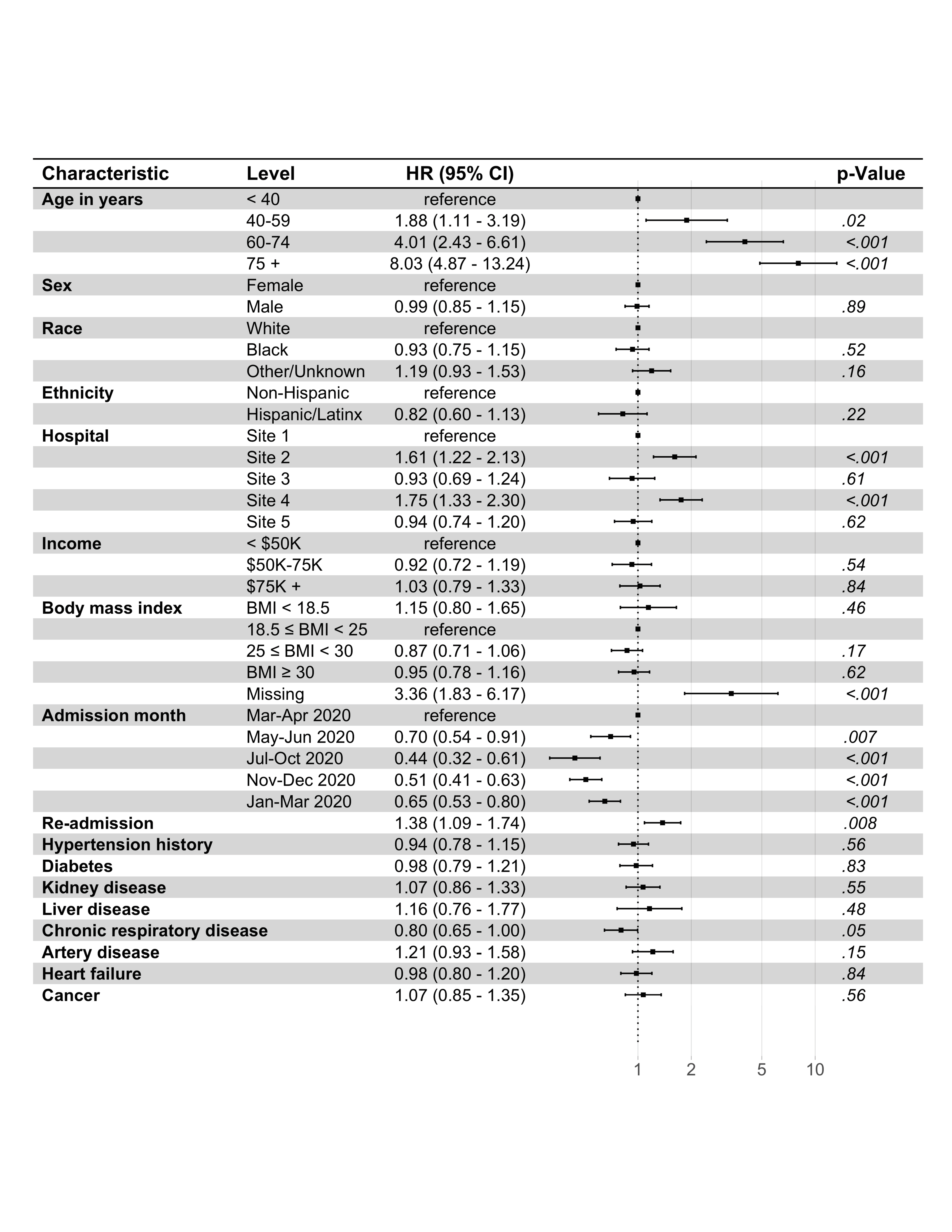

Supplement: S5 Fig — N = 6240. 15 observations deleted due to missingness in median household income. ICU Stay: Indicator for whether a patient spent any time in the ICU during first hospital stay; CI: Confidence interval; Site 1 (n = 1762), Site 2 (n = 1048), Site 3 (n = 1030), Site 4 (n = 1163), Site 5 (n = 1252) are unique hospitals in the University of Pennsylvania Health System. (TIF) [file pone.0268528.s009.tif]

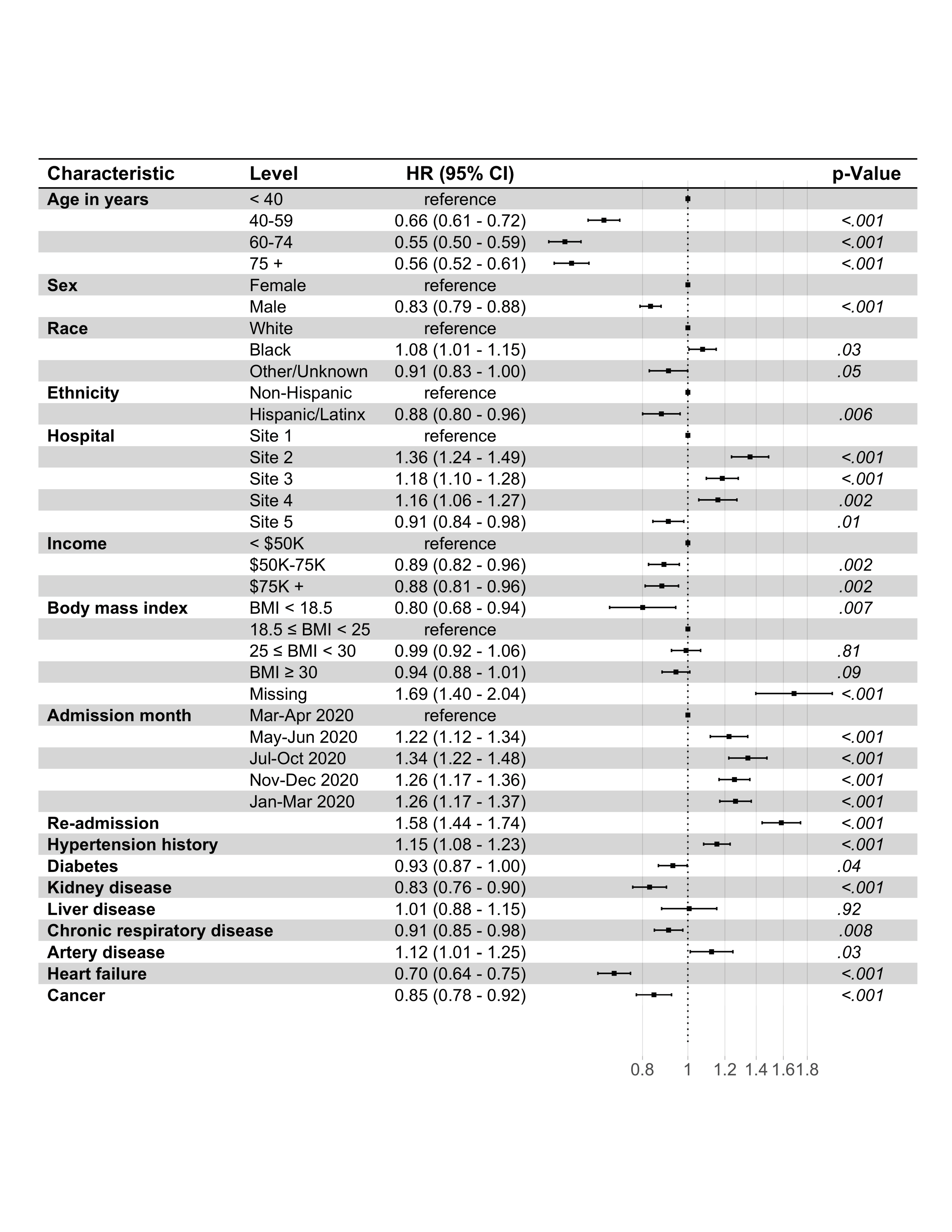

Supplement: S6 Fig — N = 6240; 15 observations deleted due to missingness in median household income. BMI: Body mass index; CI: Confidence interval; Site 1 (n = 1762), Site 2 (n = 1048), Site 3 (n = 1030), Site 4 (n = 1163), Site 5 (n = 1252) are unique hospitals in the University of Pennsylvania Health System. (TIF) [file pone.0268528.s010.tif]

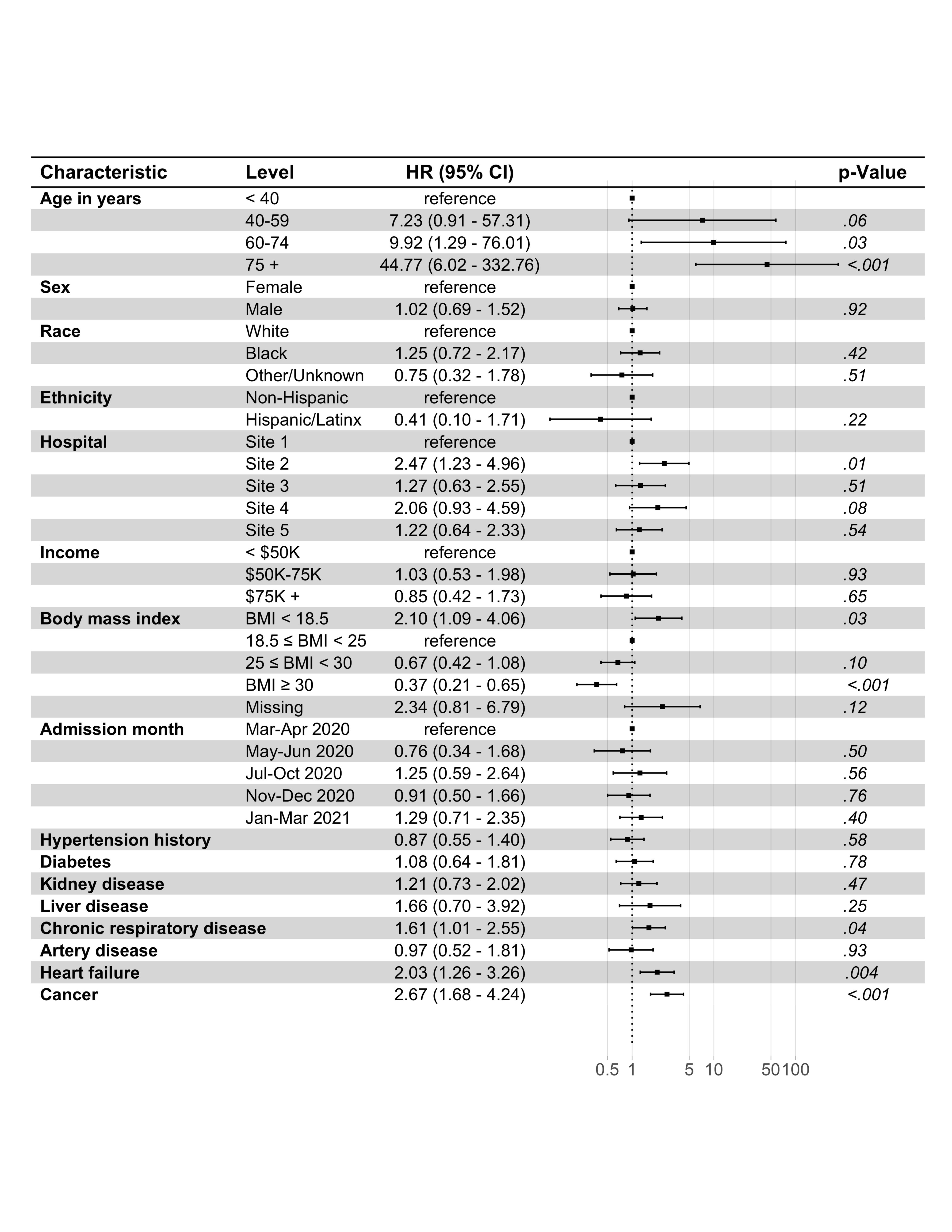

Supplement: S7 Fig — N = 6240; 15 observations deleted due to missingness in median household income. BMI: Body mass index; CI: Confidence interval; Site 1 (n = 1762), Site 2 (n = 1048), Site 3 (n = 1030), Site 4 (n = 1163), Site 5 (n = 1252) are unique hospitals in the University of Pennsylvania Health System. (TIF) [file pone.0268528.s011.tif]

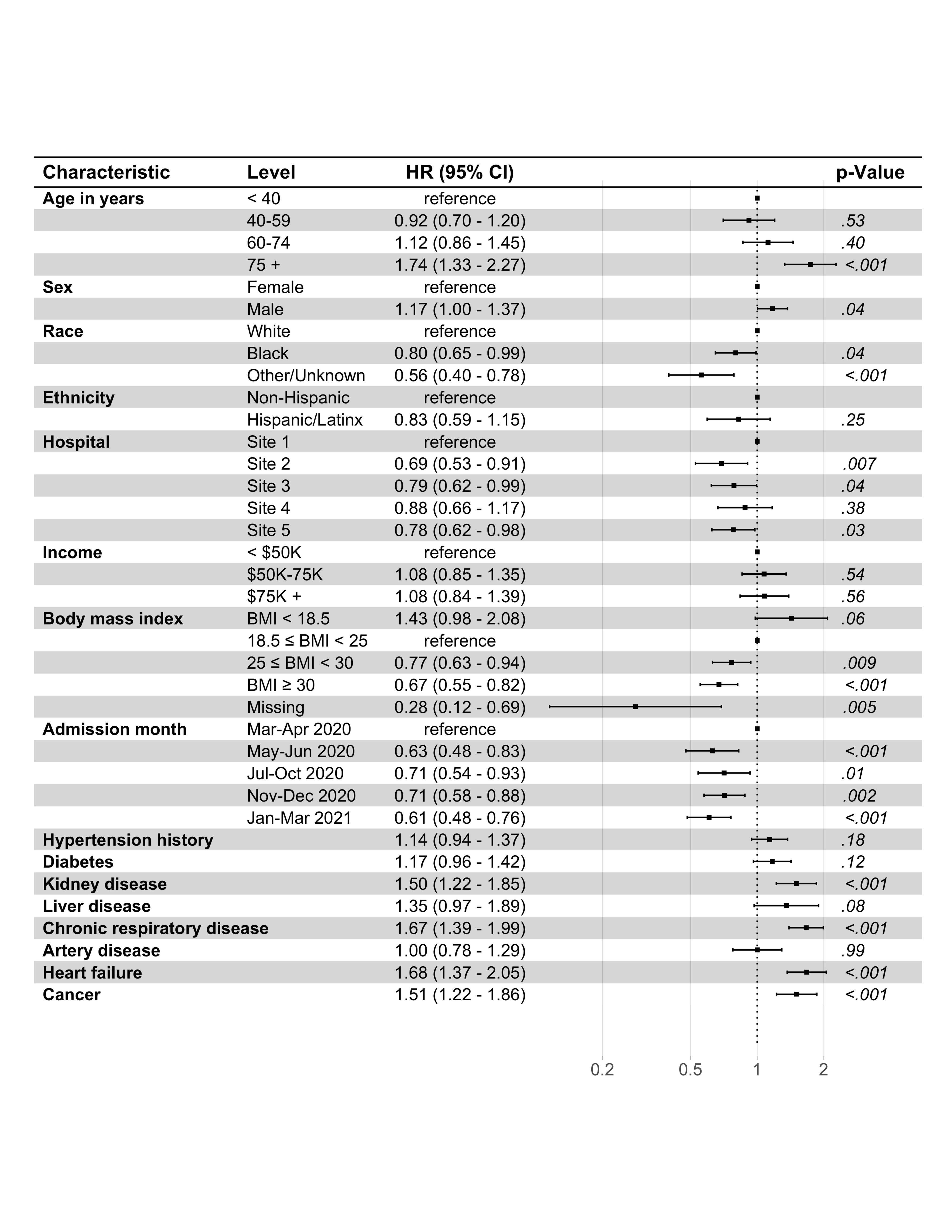

Supplement: S8 Fig — N = 6240; 15 observations deleted due to missingness in median household income. BMI: Body mass index; CI: Confidence interval; Site 1 (n = 1762), Site 2 (n = 1048), Site 3 (n = 1030), Site 4 (n = 1163), Site 5 (n = 1252) are unique hospitals in the University of Pennsylvania Health System. (TIF) [file pone.0268528.s012.tif]

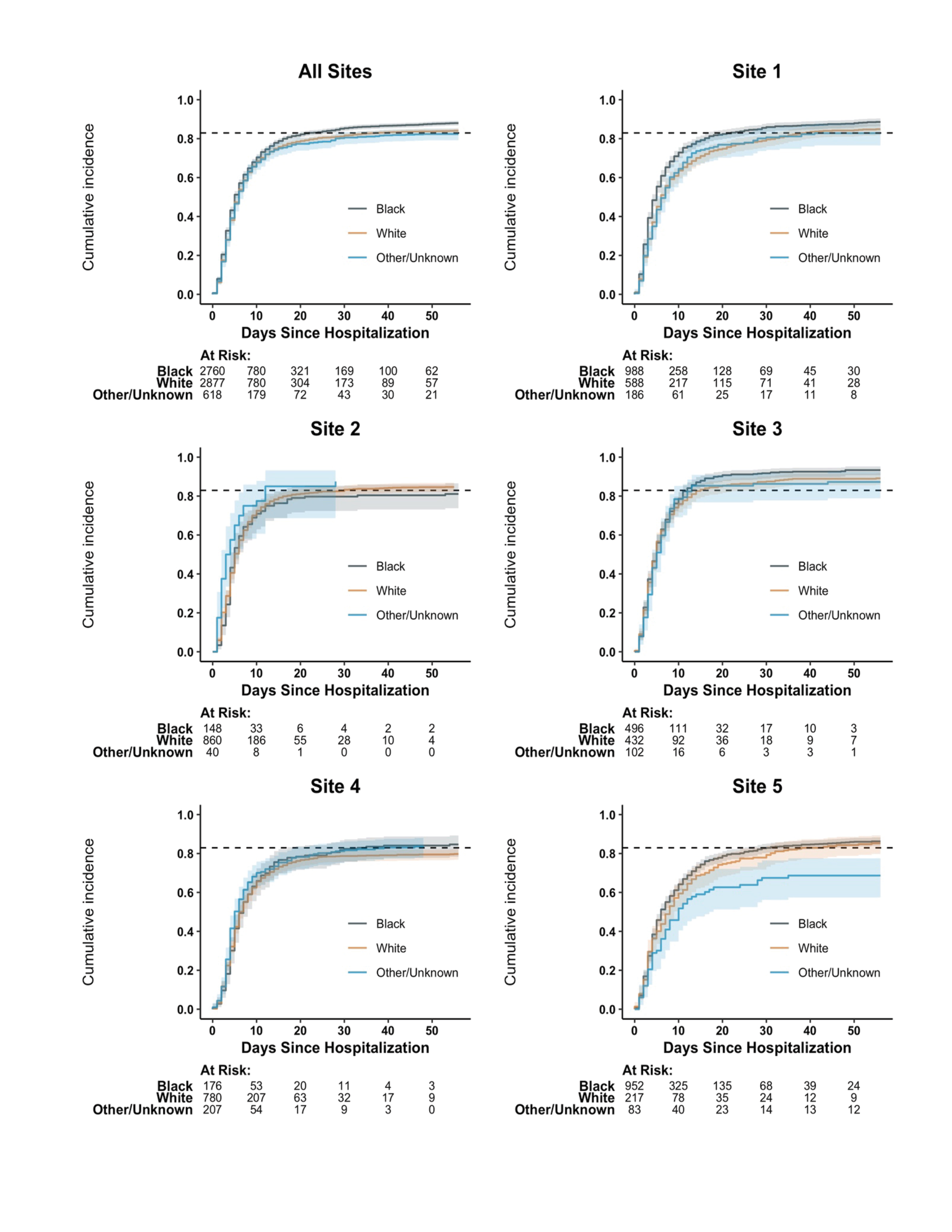

Supplement: S9 Fig — N = 6255. Site 1 (n = 1762), Site 2 (n = 1048), Site 3 (n = 1030), Site 4 (n = 1163), Site 5 (n = 1252) are unique hospitals in the University of Pennsylvania Health System. (TIF) [file pone.0268528.s013.tif]
